# Shifting trends in bacteriology and antimicrobial resistance among gastrointestinal fistula patients in China: an eight-year review in a tertiary-care hospital

**DOI:** 10.1186/s12879-017-2744-7

**Published:** 2017-09-21

**Authors:** Qinjie Liu, Jianan Ren, Xiuwen Wu, Gefei Wang, Zhiwei Wang, Jie Wu, Jinjian Huang, Tianyu Lu, Jieshou Li

**Affiliations:** 1 Department of Surgery, Jinling Hospital, Nanjing Medical University, 305 East Zhongshan Road, Nanjing, 210002 People’s Republic of China; 2 Department of Surgery, Jinling Hospital, Medical School of Nanjing University, 305 East Zhongshan Road, Nanjing, People’s Republic of China; 3 Department of Surgery, Jinling Hospital, Medical School of Southeast University, 305 East Zhongshan Road, Nanjing, People’s Republic of China

**Keywords:** Bacteriology, Antibiotic resistance, Intra-abdominal infections

## Abstract

**Background:**

The purpose of this study was to determine the shifting trends in bacteriology and antimicrobial resistance of infectious specimens isolated from gastrointestinal (GI) fistula patients over eight years in China.

**Methods:**

We retrospectively reviewed the microbial records of intra-abdominal specimens at a teaching hospital from 2008 to 2015. Study period was divided into the first half (2008–2011) and the second half (2012–2015). All isolates underwent antibiotic susceptibility testing by the micro dilution method.

**Results:**

A total of 874 intra-abdominal isolates were consecutively collected from 502 GI fistula patients (mean age, 46.5 years, 71.1% male) during the study period. Patients in the second study period (2012–2015) were older (>65 years) and more likely to have experienced cancer. Over the entire study period, most infections were caused by *E. coli* (24.2%) and *K. pneumonia* (14.1%). There was a significant decrease in the proportion *E. coli* isolates that were extended- spectrum beta-lactamase (ESBL)-positive (*P* = 0.026). The proportion of *E. coli* resistant to imipenem increased from 14.3% in 2008–2011 to 25.9% in 2012–2015 (*P* = 0.037). Imipenem resistance prevalence was higher in ESBL-negative bacteria than ESBL-positive bacteria for both *E. coli* and *K. pneumonia* (*P* < 0.001). In *Enterococcus*, significant increase in resistance to ampicillin (*P* = 0.01) and moxifloxacin (P = 0.02) over time were observed. In *Staphylococcus* and fungi, rates of antibiotic resistance did not significantly change over the study period.

**Conclusions:**

Gram-negative bacteria predominated as causative agents of intra-abdominal infections in GI fistula patients, and there was an increase in levels of resistance to certain antibiotics, particularly carbapenems. Infection control and source control are important tools available to surgeons to prevent the emergence of antibiotic-resistant pathogens.

**Electronic supplementary material:**

The online version of this article (10.1186/s12879-017-2744-7) contains supplementary material, which is available to authorized users.

## Background

Gastrointestinal (GI) fistula is a complex and challenging problem associated with intra-abdominal infections (IAIs), leading to high morbidity and mortality worldwide [1, 2]. Effective treatment for IAIs patients involves both source control and antimicrobial therapy [3]. Despite improvements in patient care, therapeutic failure remains common [4].

Selection and prompt initiation of the appropriate empiric antimicrobial therapy play an important role in decreasing morbidity and mortality in GI fistula patients with IAIs [5]. The Infectious Diseases Society of America (IDSA) guidelines recommend use of single agents, such as carbapenems, piperacillin/tazobactam, cephalosporins, fluoroquinolones and aminoglycosides combined with metronidazole to treat IAIs in adults [2]. The distribution of pathogens causing IAIs and their drug susceptibility profiles may change over time, particularly with the spread of antibiotic resistance, making it more challenging for surgeons to select appropriate antibiotic therapies [6, 7]. To improve the outcome of GI fistula patients with IAIs, it is essential for surgeons to be aware of the local bacteriology and antimicrobial resistance trends of the causative pathogens [8].

Large-scale antibiotic susceptibility surveillances have been launched over the past decades which have informed surgeons of current trends in the emergence of antibiotic-resistant bacterial strains involved in IAIs [9–11]. However, these short-term surveillances might put up an incomplete facade pattern as the fluctuations of antibiotic resistance appeared in the shorter time period [12]. Therefore, a longitudinal surveillance is critical as guidance for empiric therapy.

## Methods

### Patients and samples

Microbiology and antibiotic susceptibility of isolates collected at Jinling Hospital between 2008 and 2015 were retrospectively reviewed using the hospital medical record system. Data extracted from the system for each isolate included demographic characteristics of the patient (age, sex), co-morbidities (hypertension, diabetes, cancer, inflammatory bowel disease, lung injury, renal injury) and fistula location. Upper GI fistula was defined as fistula located in the stomach or duodenum and lower GI fistula was defined as fistula located in the jejunum, ileum or colon [13]. Isolates from tissue, fluid or deep wound cultures obtained during operation, abdominal drains, fluid from paracentesis or percutaneous aspiration of abscesses were included, and those from drain bottles, stool, superficial wounds, or perirectal abscess were excluded.

The study protocol was approved by the Institutional Review Board Ethics Committee of Jinling Hospital, and all research work was in compliance with the Helsinki Declaration.

### Pathogenic examination and antibiotic susceptibility determination

Samples were collected with sterile cotton swabs (Zhejiang Gongdong Medical Technology Co. Ltd., Taizhou, Zhejiang, China) and then sent to the microbiology laboratory for processing. Bacteria were isolated and then identified by the Vitek and Analytical Profile Index (API) bacterial identification systems or by traditional manual methods (BioMérieux, Hazelwood, MO, USA).

To assess antimicrobial susceptibility, minimum inhibitory concentrations (MICs) for each antimicrobial agent were determined by the agar dilution method, according to each year’s CLSI guidelines (Clinical Laboratory Standards Institute, USA, as annually updated) [14]. Phenotypic identification of extended-spectrum beta-lactamase (ESBL) production of *Escherichia. Coli* (*E. coli)*, *Klebsiella* and *Enterobacter species* were expanded. If MICs of ceftazidime, cefepime, or ceftriaxone were ≥2 mg/L among *E. coli, Klebsiella* or *Enterobacter species*, ESBL production was suspected. For these ESBL-suspected isolates, if the MIC of cefepime was at least eightfold more than that of cefepime in the presence of clavulanic acid, ESBL production was identified [15]. *Escherichia coli* ATCC 25922, *Klebsiella pneumonia* ATCC 700603 and *Pseudomonas aeruginosa* ATCC 27853 were used as quality control strains.

### Statistical analysis

Descriptive statistics were presented for categorical variables and continuous variables. We divided the study period into two periods for analysis: 2008–2011 and 2012–2015. We use the Mantel–Haenszel linear-by-linear association χ^2^ test to detect significant differences over time. Continuous variables were analyzed using the student t-test. *P* < 0.05 was considered statistically significant. All statistical analyses were performed using SPSS software (Version 22 IBM, Armonk, NY).

## Results

### Patient characteristics

A total of 502 GI fistula patients (mean age 46.5 years, 71.1% male) were included. Demographic characteristics of included patients are shown in Table1. Patients in the second study period (2012–2015) exhibited significant enrichment of clinical factors, including advanced age (*P* = 0.02), cancer (16.0% VS 6.7%, *P* = 0.001) and renal injury (16.7% VS 9.2%, *P* = 0.013) than patients from 2008 to 2011. In addition, the 2012–1015 cohort had a significantly higher percentage of lower GI fistula (*P* = 0.005) and a lower percentage of intensive care unit (ICU) patients (*P* < 0.001) (Table 1). We did not find the difference change in mortality rates over time (27.6% VS 28.1%, *P* = 0.896).

**Table 1:** Clinical characteristics of patients during 2008 and 2015

	2008–2012 (*n* = 239)	2012–2015 (*n* = 263)	*P*
Gender			
Male	169 (70.71%)	188 (71.48%)	0.849
Age (years)	44.85 ± 14.99	48.05 ± 15.62	0.020
≤ 16	6 (2.51%)	3 (1.14%)	0.248
17–32	49 (20.50%)	41 (15.59%)	0.152
33–48	88 (36.82%)	96 (36.50%)	0.941
49–64	76 (31.80%)	81 (30.80%)	0.809
≥ 65	20 (8.37%)	42 (15.97%)	0.010
Patient location			
ICU	150 (62.76%)	123 (46.77%)	<0.001
Fistula location,			
Upper gastrointestinal	107 (45.15%)	98 (37.26%)	0.073
Lower gastrointestinal	106 (44.35%)	150 (57.03%)	0.005
Both	19 (7.95%)	15 (5.70%)	0.317
Co-morbidities			
Hypertension	39 (16.32%)	57 (21.67%)	0.128
Diabetes	20 (8.37%)	30 (11.41%)	0.256
Cancer	16 (6.69%)	42 (15.97%)	0.001
IBD	8 (3.35%)	16 (6.08%)	0.151
Lung Injury	33 (13.81%)	45 (17.11%)	0.308
Renal Injury	22 (9.21%)	44 (16.73%)	0.013
30-day mortality	66 (27.62%)	74 (28.14%)	0.896

### Microbiological profile

**Fig. 1:**
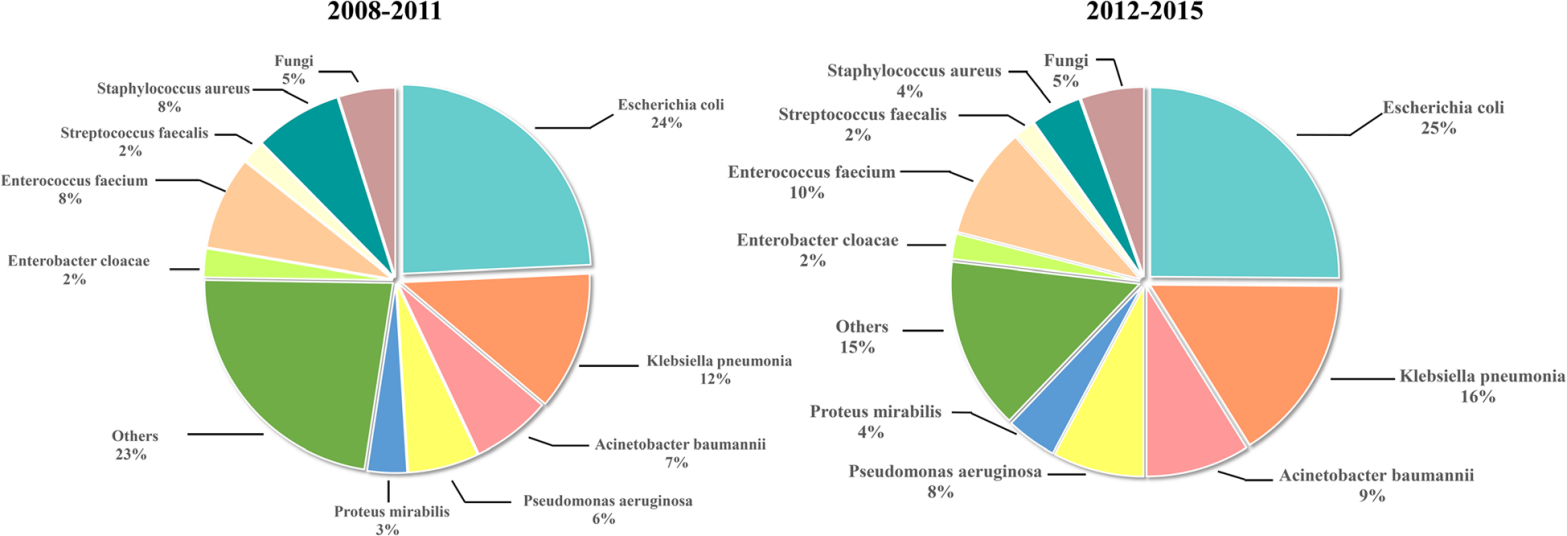
Distribution of strain groups identified in the study periods

During the entire study period, 874 isolates were collected, and the mean number of isolates per year was 109+/−19. Co-infection with multiple microbial strains was identified in 118(49.4%) patients during the first study period and 124(47.2%) during the second period. The distribution of microbial strains, stratified by study period, is shown in Fig. 1. The total number of Gram-negative was 638 (73.0%), which became more common over time (*P* = 0.024), followed by Gram-positive isolates (188, 25.5%), respectively. Overall, *E. coli* was the most frequently identified bacterial microorganism (216 isolates, 24.2% of all bacterial growths and 33.9% of Gram-negative isolates), followed by *K. pneumonia* (123 isolates, 14.1% of all bacterial growths and 19.3% of Gram-negative isolates). A significant decrease in the percentage of *E. coli* isolates that were ESBL-positive occurred between study periods (*P* = 0.026), but there was no significant difference in the proportion of *K. pneumonia* that were ESBL positive between study periods. The common Gram-positive bacteria were *Enterococcus* and *Staphylococcus*. (Additional file 1: Table S1).

### *Enterobacteriaceae* antibiotic resistance

Tables 2 and 3 list the in vitro resistance profiles of *E. coli* and *K. pneumonia,* respectively, stratified by ESBL-production ability. There were similar patterns of antibiotic resistance for *E. coli* and *K. pneumonia* isolates over the study period, with both displaying high levels of resistance to penicillins, cephalosporins and fluoroquinolones. There was a statistically significant decrease in prevalence of resistance to ceftazidime and cefepime in *E. coli* isolates (*P* = 0.042, *P* = 0.035). No significant change in prevalence of resistance to aztreonam and amikacin was observed in both *E. coli* and *K. pneumonia* isolates. Resistance to amikacin was relatively low compared with the other antibiotics mentioned above for both *E. coli* and *K. pneumonia* isolates.

**Table 2:** Antimicrobial resistance of *Escherichia coli* isolates to the tested antibiotics

Resistance rate (%)				
Isolate/Antibiotics	2008–2011	2012–2015	Total	P for Trend Test
All *Escherichia coli*				
AMK	7.78	14.16	11.33	0.154
AMP	97.92	96.49	97.14	0.537
SAM	81.97	87.93	85.88	0.279
TZP	23.47	25.00	24.30	0.795
CZO	92.55	91.30	91.87	0.742
CAZ	81.82	69.83	75.35	**0.042**
FEP	75.51	62.07	68.22	**0.035**
IPM	14.29	25.86	20.56	**0.037**
CIP	90.16	79.13	82.95	0.064
ESBL+				
AMK	6.78	12.33	9.85	0.287
AMP	100.00	100.00	100.00	–
SAM	77.78	93.15	88.07	**0.020**
TZP	20.31	6.85	13.14	**0.020**
SXT	84.21	73.61	77.27	0.207
CZO	100.00	100.00	100.00	–
CAZ	89.23	68.49	78.26	**0.003**
FEP	81.25	60.27	70.07	**0.007**
IPM	12.31	8.22	10.14	0.427
CIP	86.11	80.82	82.57	0.494
ESBL-				
AMK	6.67	16.22	13.46	0.361
AMP	88.24	89.74	89.29	0.867
SAM	77.78	77.50	77.55	0.986
ZP	35.29	57.50	50.88	0.125
SXT	88.89	67.50	71.43	0.199
CZO	58.82	74.36	69.64	0.245
CTT	28.57	70.00	63.83	**0.035**
CAZ	52.94	70.00	64.91	0.217
FEP	52.94	62.50	59.65	0.501
IPM	43.75	57.50	53.57	0.351
CIP	88.89	77.50	79.59	0.444
Not all tested antibiotics are listed				

*AMK* Amikacin, *AMP* Ampicillin, *SAM* Ampicillin/Sulbactam, *CAZ* Ceftazidime, efepime, *CTT* Cefotetan, *CZO* Cefazolin, *IPM* Imipenem, *TZP* Piperacillin-Tazobactam, *CIP* Ciprofloxacin

Data in boldface reflected p values < 0.05

**Table 3:** Antimicrobial resistance of *Klebsiella pneumonia* isolates to the tested antibiotics

Resistance rate (%)				
Isolate/Antibiotics	2008–2011	2012–2015	Total	P for Trend Test
All *Klebsiella pneumonia*				
AMK	40.91	36.11	37.93	0.605
SAM	97.14	85.14	88.99	0.061
TZP	75.00	58.11	64.75	0.056
CTT	60.61	54.79	56.60	0.576
CAZ	89.80	78.38	82.93	0.099
FEP	75.00	67.57	70.49	0.379
IPM	65.31	56.76	60.16	0.343
CIP	74.29	75.68	75.23	0.875
ESBL+				
AMK	33.33	7.41	16.67	**0.031**
SAM	100.00	89.29	92.11	0.281
TZP	55.56	17.86	32.61	**0.008**
CTT	12.50	14.81	14.29	0.869
CAZ	94.44	71.43	80.43	0.055
FEP	83.33	53.57	65.22	**0.039**
IPM	38.89	14.29	23.91	0.056
CIP	50.00	71.43	65.79	0.220
ESBL-				
AMK	40.91	53.33	49.25	0.339
SAM	94.44	82.61	85.94	0.221
TZP	86.36	82.61	83.82	0.694
CTT	88.89	78.26	81.25	0.327
CAZ	82.61	82.61	82.61	–
FEP	72.73	76.09	75.00	0.765
PM	78.26	82.61	81.16	0.663
CIP	83.33	78.26	79.69	0.650
Not all tested antibiotics are listed				

*AMK* Amikacin, *SAM* Ampicillin/Sulbactam, *CAZ* Ceftazidime, *FEP* Cefepime, *CTT* Cefotetan, *IPM* Imipenem, *TZP* Piperacillin-Tazobactam, *CIP* Ciprofloxacin

Data in boldface reflected p values < 0.05

In ESBL-positive *E. coli*, prevalence of resistance to piperacillin/tazobactam (13.1%) was lower than ampicillin/sulbactam resistance prevalence (88.1%) and both decreased over the study period (*P* = 0.020). The same resistance trend to piperacillin/tazobactam was also observed in ESBL-positive *K. pneumonia* (*P* = 0.008). Imipenem resistance prevalence was higher in *K. pneumonia* than *E. coli.* It increased over time in *E. coli* (14.3% VS 25.9%, *P* = 0.037) but there was no significant change in *K. pneumonia* (65.3% VS 56.8%, *P* = 0.343). Imipenem resistance prevalence was higher in ESBL-negative than ESBL-positive bacteria for both *E. coli* and *K. pneumonia* (*P* < 0.001).

### Antimicrobial resistance of non-fermenting bacteria

In *A. baumannii* isolates, extremely high levels of cephalosporin resistance were observed, which increased to 100% in 2012–2015. In contrast, ceftazidime and cefepime resistance rates were lower in *P. aeruginosa* isolates (Table 4). Both *A. baumannii* and *P. aeruginosa* showed strikingly high resistance rates to imipenem during the study period. Among *A. baumannii* isolates*,* imipenem resistance prevalence was 95.6% and did not significantly change during the study period. In contrast, imipenem resistance in *P. aeruginosa* isolates was much lower. Resistance to fluoroquinolones showed no significant change over time in either pathogen.

**Table 4:** Antimicrobial resistance of *Acinetobacter baumannii* and *Pseudomonas aeruginosa* isolates to the tested antibiotics

Resistance rate (%)				
Isolate/Antibiotics	2008–2011	2012–2015	Total	P for Trend Test
Acinetobacter baumannii				
AMP	96.43	100.00	98.55	0.223
SAM	90.48	100.00	96.77	**0.045**
TZP	96.30	95.00	95.52	0.801
SXT	95.24	73.17	80.65	**0.037**
CRO	94.44	100.00	98.31	0.128
CTX	100.00	100.00	100.00	–
CAZ	88.89	100.00	95.59	**0.029**
FEP	96.30	100.00	98.53	0.214
IPM	96.30	95.12	95.59	0.818
LVX	66.67	56.10	60.29	0.383
CIP	95.24	97.56	96.77	0.624
Pseudomonas aeruginosa				
AMK	31.82	22.22	25.86	0.418
SAM	100.00	100.00	100.00	–
TZP	60.87	33.33	44.07	**0.038**
ATM	58.82	75.00	61.90	0.549
SXT	100.00	97.22	98.11	0.488
CRO	100.00	100.00	100.00	–
CTX	100.00	100.00	100.00	–
CAZ	69.57	61.11	64.41	0.508
FEP	65.22	50.00	55.93	0.251
IPM	73.91	58.33	64.41	0.223
LVX	34.78	44.44	40.68	0.461
CIP	38.89	41.18	40.38	0.873
Not all tested antibiotics are listed				

*AMK* Amikacin, *SAM* Ampicillin/Sulbactam, *TZP* piperacillin-tazobactam, *ATM* Aztreonam, *SXT* Trimethoprim/Sulfamethoxazole, *CRO* Ceftriaxone, *CTX* cefotaxime, *CAZ* ceftazidime, *FEP* cefepime, *IPM* imipenem, *LVX* levofloxacin, *CIP* ciprofloxacin

Data in boldface reflected p values < 0.05

### Antimicrobial resistance of gram-positive bacteria and fungi

Antibiotic resistance prevalence rates of Gram-positive bacteria are listed in Table 5. In *Enterococcus*, resistance to ampicillin increased from 72.2% in 2008–2011 to 92.5% in 2012–2015 (*P* = 0.01). Resistance to moxifloxacin also increased significantly (*P* = 0.02). No significant changes in resistance to vancomycin (*P* = 0.311) and linezolid (*P* = 0.111) over time were observed.

**Table 5:** Antimicrobial resistance of *Enterococcus* and *Staphylococcus* isolates to the tested antibiotics

Resistance rate (%)				
Isolate/Antibiotics	2008–2011	2012–2015	Total	P for Trend Test
Enterococcus				
AMP	72.22	92.45	84.27	**0.01**
STH	47.06	57.58	52.24	0.389
GEH	71.74	79.25	75.76	0.385
ERY	88.57	88.68	88.64	0.988
CIP	89.29	90.57	90.12	0.854
CLI	100.00	95.56	95.92	0.667
MFX	63.64	91.11	85.71	**0.02**
PEN	87.23	92.31	89.90	0.403
TCY	74.29	69.23	71.26	0.609
VAN	4.26	9.43	7.00	0.311
LNZ	4.88	0.00	2.17	0.111
LVX	77.14	90.38	85.06	0.089
Staphylococcus				
OXA	82.69	93.10	86.42	0.190
SXT	47.62	24.14	38.03	0.045
ERY	84.62	82.76	83.95	0.827
CIP	73.68	89.66	83.33	0.147
CLI	69.57	57.14	64.86	0.278
MFX	46.34	37.93	42.86	0.484
PEN	94.34	100.00	96.34	0.192
GEN	71.15	72.41	71.60	0.904
TCY	56.82	68.97	61.64	0.296
VAN	0.00	0.00	0.00	–
LNZ	0.00	0.00	0.00	–
LVX	68.89	79.31	72.97	0.324
Not all tested antibiotics are listed				

*AMP* Ampicillin, *OXA* Oxacillin, *STH* Streptomycin-High, *SXT* Trimethoprim/Sulfamethoxazole, *ERY* erythromycin, *GEH* gentamicin, *CIP* ciprofloxacin, *CLI* Clindamycin, *MFX* moxifloxacin, *PEN* penicillin, *TCY* tetracycline, *VAN* Vancomycin, *LNZ* Linezolid, *LVX* Levofloxacin

Data in boldface reflected p values < 0.05

Methicillin-resistant *S. aureus* (MRSA) accounted for 94.1% of *S. aureus* isolates in 2012–2015. There was no significant change in *S. aureus* resistance to oxacillin over time. All of 51 *Staphylococcus* were susceptible to vancomycin (Table 5).

Fungi isolates showed lower antibiotic resistance rates than bacteria isolates, and rates did not significantly vary over time (Table 6).

**Table 6:** Antimicrobial resistance of *Fungi* isolates to the tested antibiotics

Resistance rate (%)				
Antibiotics	2008–2011	2012–2015	Total	P for Trend Test
FLU	18.18	8.33	11.43	0.395
VOR	0.00	8.33	5.71	0.324
ITR	18.18	5.56	10.34	0.279

*FLU* Fluconazole, *VOR* Voriconazole, *ITR* Itraconazole

## Discussion

To our knowledge, this is the first study to examine the shifting trends in bacteriology and antimicrobial resistance among GI fistula patients in China. Our findings indicate a significant increase in the percentage of IAIs attributable to Gram-negatives bacteria, with a corresponding decrease in the percentage attributable to Gram-positive isolates. There was a trend for increased resistance prevalence levels to certain antibiotics for Gram-negative bacteria, especially carbapenems.

*K. pneumonia* and *A. baumannii* have gained notoriety as important pathogens because of their increasing resistance to antibiotics and a rise in the number of severe infections caused by these micro-organisms in surgical settings [16]. We found an increase in IAIs attributable to *K. pneumonia* and *A. baumannii* infection over time, although this increase did not reach statistical significance. Colonization with these bacteria have been described as the reason for high incidence in surgical wards and this could be prevented through effective infection control [17, 18]. Therefore, we must heighten our awareness of the importance of infection control.

ESBL production which can hydrolyze β-lactam antibiotics has been increasingly identified worldwide amongst the *Enterobacteriaceae* family, particularly *E. coli* and *K. pneumonia* [19]. In the present study, the overall prevalence of ESBL-positive strains of *E. coli* was 63.9%, which decreased significantly over time, and the overall prevalence of ESBL-positive strains of *K. pneumonia* was 37.3%, which did not significant change over the study periods. These levels are somewhat lower than those reported by SMART research in 2012 and 2013 [10]. Carbapenems and piperacillin-tazobactam are the most potent and reliable antibiotics for the treatment of ESBL-producing infection [20]. In our study, we found that resistance to piperacillin-tazobactam decreased over time both ESBL-producing *E. coli* (*P* = 0.02) and *K. pneumonia* (*P* = 0.008). It suggests that piperacillin-tazobactam is a suitable treatment option for these infections [21].

Resistance to carbapenems is associated with high mortality and has been an emerging concern worldwide [22, 23]. The overall prevalence of imipenem resistance in *E. coli* isolates was 20.6%, which significantly increased over time. Prevalence in *K. pneumonia* was 60.2%, which did not change over time. Both these prevalence levels are higher than previous reports [9, 24–26]. This may be because the majority of our patients have transferred from other hospitals and have been treated with antibiotics for a number of days, which has been shown to be a risk factor for carbapenem resistance [27]. High resistance prevalence has also resulted from its spread in surgical wards and ICUs [12]. Standard infection control practice (basic hand hygiene, active surveillance cultures of patients, staff, and the environment) should be carried out to prevent the colonization and spread of resistant bacteria [8, 28, 29].

The prevalence of multidrug resistance amongst *A. baumannii* isolates makes carbapenem the most effective treatment [30]. Carbapenem resistance has become a serious problem, with prevalence reaching a remarkable 95.6% of all isolates in our study. Similarly high levels have been reported in blood stream infections [23]. Once carbapenem resistant *A. baumannii* emerges, the infected patient has little chance of effective treatment [31]. Therefore, we need to pay attention to source control and limiting the spread of carbapenem-resistant bacteria.

*P. aeruginosa* is another a common Gram-negative non-fermenting pathogen causing IAIs. In this study, the most efficient antimicrobial agent for *P. aeruginosa* was found to be amikacin, as has been reported elsewhere [32]. However, we rarely treat patients with amikacin because of its renal toxicity. In our study we observed a significant decrease in resistance to piperacillin-tazobactam over time, suggesting that piperacillin- tazobactam could be the first choice treatment option for patients infected by *P. aeruginosa*, as recommended by several studies [33, 34].

The proportion of Gram-positive bacterial isolates that were *Enterococcus* increased over time. Antibiotic resistance rates for this group of pathogens also increased. *Staphylococcus* isolates had high levels of penicillin G, macrolide, and clindamycin resistance, but no resistance to vancomycin or linezolid was observed. Antibiotics resistance levels were lower among Gram-positive than Gram-negative bacteria. we therefore recommend focusing on Gram-negative bacteria with high antibiotic resistance in GI fistula patients.

In an attempt to identify factors that might influence antibiotic resistance emergence, we analyzed the clinical characteristics of patients. We found that patients in the second study period were older (aged >65 years) and were more likely to suffer cancer, both of which have been demonstrated as risk factors for antibiotic resistance [8, 18, 35]. We also found more IAIs caused by lower GI fistula in the second study period. A recent study by *Mu* et al. reported that antibiotic intervention exerts location-specific effects on antibiotic resistance genes (increased in the lower GI tract) [36]. Most of our patients were transferred from other hospitals, which means they had been previously treated with antibiotics and were therefore at increased risk of antibiotic resistance. Excessive antibiotic use has been linked with the development of resistance, which is a common practice in many developing countries [18]. Combined, these factors at least partly explain the increase in antibiotic resistance that we have observed. We found that smaller ICU patients showed higher antibiotic resistance. And that again underlines the serious antibiotic resistance.

Inappropriate use of antibiotics and inadequate source control were found to be independent predictors of mortality in a previous analysis [37]. High levels of antibiotic resistance have left few treatment options available to surgeons [8]. However, we found no change in mortality rates over time. This could partially be attributable to the effective management of source control. Newer IAI treatment guidelines recommend intravenous antimicrobial agents as a supplement to source control, and source control may be an available option for surgeons to prevent the emergence of antibiotic-resistant microbial strains [38].

There are some limitations to our study. First, it is a retrospective and single-center surveillance study, which may explain the higher resistance levels observed in our study than other reports from China [23]. However, the critically ill patients at our center were transferred from other hospitals throughout the country, so our study may represent the bacteriology and antimicrobial resistance profiles of severely infected GI fistula patients in China more generally. Second, we did not perform polymerase chain reaction (PCR) and DNA sequencing of isolates. Third, we did not use the unified CLSI breakpoints, as annually updated. In fact, our microbiology laboratory updated determinations according to the newest CLSI documents and 2008–2015 isolates were determined by each year’s documents. Change of breakpoints might cause fluctuations of antimicrobial resistance in short-term surveillances [12]. But there is not a large difference between CLSI breakpoints. And a longitudinal surveillance spanning over 8 years is of great significance for monitoring resistance, which may minimize referral bias.

## Conclusions

This study illustrates the shifting trends in bacteriology and antimicrobial resistance in GI fistula patients in China over time. Gram-negative bacteria have become a more significant cause of IAIs in these patients. Currently, carbapenem resistances in Gram-negative bacteria is a serious problem in this patient group. Our findings confirm the urgent need to continue surveillance studies that monitor bacteriology and antimicrobial resistance trends. Infection control and source control are important tools for surgeons to use to prevent the emergence of isolated antibiotic-resistant pathogens.

## Additional file

Additional file 1: Table S1. Bacterial identification of isolates from intra-abdominal infections in a Tertiary-Care Hospital during 2008 and 2015. (DOCX 15 kb)

## Abbreviations

AMK: Amikacin
AMP: Ampicillin
ATCC: American Type Culture Collection
ATM: Aztreonam
CAZ: Ceftazidime
CIP: Ciprofloxacin
CLI: Clindamycin
CLSI: Clinical Laboratory Standards Institute
CRO: Ceftriaxone
CTT: Cefotetan
CTX: Cefotaxime
CZO: Cefazolin
ERY: Erythromycin
ESBL: extended-spectrum beta-lactamase
FEP: Cefepime
FLU: Fluconazole
GEH: Gentamicin
GEN: Gentamicin
GI fistula: gastrointestinal fistula
IAIs: intra-abdominal infections
IDSA: Infectious Diseases Society of America
IPM: Imipenem
ITR: Itraconazole
KPC: Carbapenem-resistant *K. pneumonia*
LNZ: Linezolid
LVX: Levofloxacin
MFX: Moxifloxacin
MICs: minimum inhibitory concentrations
MRSA: Methicillin-resistant *S. aureus*
OXA: Oxacillin
PCR: Polymerase chain reaction;
PEN: Penicillin G
QDA: Quinupristin/Dalfopristin
SAM: Ampicillin/Sulbactam
SMART: Study for Monitoring Antimicrobial Resistance Trends
STH: Streptomycin-High
SXT: Trimethoprim/Sulfamethoxazole
TCY: Tetracycline
TOB: Tobramycin
TZP: Piperacillin/Tazobactam
VAN: Vancomycin
VOR: Voriconazole
