# Supplementary material for: Shifting trends in bacteriology and antimicrobial resistance among gastrointestinal fistula patients in China: an eight-year review in a tertiary-care hospital
Source: BMC Infect Dis. 2017 Sep 21;17:637. doi: 10.1186/s12879-017-2744-7 (PMC5609055; doi:10.1186/s12879-017-2744-7)
Supplement: Additional file 1: Table S1. — Bacterial identification of isolates from intra-abdominal infections in a Tertiary-Care Hospital during 2008 and 2015. (DOCX 15 kb) [file 12879_2017_2744_MOESM1_ESM.docx]

Additional file 1: Table S1. Bacterial identification of isolates from intra-abdominal infections in a Tertiary-Care Hospital during 2008 and 2015.

|  | **2008-2011** | **2012-2015** | **Total** |  |
| --- | --- | --- | --- | --- |
| **Bacteria** | **n (%)** | **n (%)** | **n (%)** | **P for Trend Test** |
| **Gram-negative bacteria** | 286(69.4) | 352(76.19) | 638(73.00) | **0.024** |
| *Escherichia coli* | 100(24.27) | 116(25.11) | 216(24.21) | 0.775 |
| ESBL-producing strains | 65(65.00) | 73(62.93) | 138(63.89) | **0.026** |
| Non-ESBL strains | 17(17.00) | 40(34.48) | 57(26.39) | - |
| Not identified | 18(18.00) | 3(2.59) | 21(9.72) | - |
| *Klebsiella pneumonia* | 49(11.89) | 74(16.02) | 123(14.07) | 0.080 |
| ESBL-producing strains | 18(36.73) | 28(37.84) | 46(37.30) | 0.525 |
| Non-ESBL strains | 23(46.94) | 46(62.16) | 69(56.10) | - |
| Not identified | 8(16.33) | 0 | 8(6.50) | - |
| *Acinetobacter baumannii* | 28(6.8) | 41(8.87) | 69(7.89) | 0.255 |
| *Pseudomonas aeruginosa* | 25(6.07) | 36(7.79) | 61(6.98) | 0.318 |
| *Proteus mirabilis* | 14(3.4) | 20(4.33) | 34(3.89) | 0.477 |
| *Enterobacter cloacae* | 10(2.43) | 10(2.16) | 20(2.29) | 0.795 |
| *Morganella morganii* | 6(1.46) | 14(3.03) | 20(2.29) | 0.120 |
| *Serratia marcescens* | 5(1.21) | 15(3.25) | 20(2.29) | **0.045** |
| *Proteus vulgaris* | 3(0.73) | 4(0.87) | 7(0.8) | 0.820 |
| **Gram-positive bacteria** | 105(25.49) | 83(17.97) | 188(21.51) | **0.007** |
| *Enterococcus faecium* | 33(8.01) | 44(9.52) | 77(8.81) | 0.431 |
| *Streptococcus faecalis* | 8(1.94) | 8(1.73) | 16(1.83) | 0.817 |
| *Staphylococcus aureus* | 31(7.52) | 20(4.33) | 51(5.84) | **0.044** |
| **Others** | 94(22.9) | 68(14.72) | 162(18.54) | **-** |
| **Fungi** | 20(4.85) | 25(5.41) | 45(5.15) | 0.710 |
